# Association Between Neonicotinoids and Neurobehavioral Development in Preschool Children from South China: A Biomonitoring-Based Study

**DOI:** 10.3390/toxics13100872

**Published:** 2025-10-14

**Authors:** Yixiang Zhou, Yong Wang, Zhan Huang, Wanting Xiao, Yujie He, Hui Guo, Wen Chen, Siqi Ai, Liecheng Hong, Lei Lu, Jianyong Lu, Chuanwen Li, Ziquan Lv, Qing Wang

**Affiliations:** 1 School of Public Health, Sun Yat-sen University, Guangzhou 510080, China; zhouyx99@mail2.sysu.edu.cn (Y.Z.);; 2 Public Health Service Center, Bao’an District, Shenzhen 518000, China; 3 Shenzhen Center for Disease Control and Prevention, Shenzhen 518055, China

**Keywords:** neonicotinoid insecticides, neurobehavioral development, cross-sectional study, vulnerable populations

## Abstract

Neonicotinoid insecticides (NEOs), one of the most widely used pesticide classes worldwide, have raised concerns due to potential neurotoxic effects. Yet evidence on human exposure and health outcomes, particularly in preschool children, remains limited. In this study, 506 children aged 3–6 years from Shenzhen, China, were assessed. Neurobehavioral development was evaluated with the Strengths and Difficulties Questionnaire (SDQ), and urinary concentrations of 11 NEOs were measured, including imidacloprid (IMI), clothianidin (CLO), thiamethoxam (THM), dinotefuran (DNT), nitenpyram (NIT), sulfoxaflor (SFX), acetamiprid (ACE), thiacloprid (THD), flonicamid (FLO), 6-chloronicotinic acid (6-CINA), N-desmethyl-acetamiprid (NACE), and N-desmethyl-thiamethoxam (NTHM). Seven compounds showed high detection rates, including IMI (97.4%), CLO (100%), THM (100%), DNT (99.8%), NIT (99.8%), NACE (100%), and NTHM (99.8%). The mean urinary concentration was 234.145 μg/g creatinine, exceeding levels in earlier studies and indicating widespread exposure. IMI, NTHM, and NACE showed significant positive dose–response relationships with emotional symptoms, hyperactivity, and total difficulties and were major contributors in mixture models; sex-stratified analyses suggested effect modification for NTHM and NACE. These findings provide new epidemiological evidence to inform public health risk assessment and regulatory action on NEOs.

## 1. Introduction

Neurobehavioral development during early childhood lays the foundation for later cognitive functioning and social adaptation. This developmental process depends heavily on tightly regulated neurobiological events such as neuronal differentiation, migration, myelination, and synaptic plasticity [1,2]. The preschool period (ages 3–6) is a particularly sensitive window, characterized by rapid structural and functional maturation of the central nervous system [3]. While neurobehavioral disorders have traditionally been attributed to genetic, psychosocial, and nutritional factors, growing evidence indicates that environmental pollutants represent an important and often underrecognized contributor, particularly when exposure occurs during early life [4,5]. During this stage, the brain is highly susceptible to external insults, and adverse environmental exposures may result in long-term or even irreversible disruptions to neurodevelopment [6]. Such exposures may disrupt neurodevelopment through mechanisms including interference with neurotransmitter systems, induction of oxidative stress, and modulation of synaptic function [7,8].

At present, several academic studies have reported associations between environmental factors such as heavy metals, organic pollutants, and air pollution with neurobehavioral abnormalities in children [9,10]. However, neonicotinoid insecticides (NEOs), as a class of emerging environmental contaminants, although their widespread presence in the environment has been well documented and animal as well as in vitro studies have demonstrated various adverse health effects including neurotoxicity, their neurotoxic impacts on human populations remain insufficiently understood and require further systematic investigation [11,12]. NEOs are synthetic insecticides structurally derived from nicotine and have been widely applied in agriculture, horticulture, and animal husbandry since the 1990s [13]. Due to their high water solubility, strong systemic activity, and environmental persistence, NEOs are widely present in surface water, groundwater, soil, agricultural products, and even the atmosphere, thereby posing potential exposure risks to humans [14,15].

Existing studies have shown that at least one neonicotinoid compound was detected in over 90% of surface water samples collected from major water bodies in China [16,17]. Moreover, neonicotinoids can be bioaccumulated in aquatic vertebrates (such as fish and amphibians) as well as invertebrates (including mayflies and water fleas), suggesting their potential transmission through the food chain and ecological threat [18]. More importantly, urine biomonitoring in some regions has shown detection rates of neonicotinoids and their metabolites approaching 100%. This indicates long-term, low-level exposure in the general population of China, with particularly elevated risks in agriculturally intensive and downstream urban areas [19]. Previous studies have demonstrated that NEOs can activate mammalian nicotinic acetylcholine receptors (nAChRs), leading to abnormal neuronal excitability and impaired synaptic formation, potentially leading to deficits in learning, memory, and behavioral regulation in animal models [20,21]. Regarding the neurotoxic mechanism of NEOs, some studies reported that exposure to NEOs may induce oxidative stress, mitochondrial dysfunction, and apoptosis, all of which are key mechanisms implicated in neurodevelopmental impairment [22,23]. Although an increasing body of evidence suggests that NEOs may exert neurotoxic effects on non-target organisms, including mammals, most of this evidence comes from laboratory studies using cell cultures or animal models. Furthermore, the NEO concentrations applied in such experiments are often several orders of magnitude higher than those encountered under real-world environmental exposure conditions [24]. Therefore, the impact of NEO exposure on human neurobehavioral development, particularly in children, requires further investigation under environmentally relevant conditions through population-based epidemiological study.

The Pearl River Delta (PRD) region in southern China provides a representative setting for such research. Due to highly intensive forestry and protected agriculture in the upper and middle reaches of the Pearl River, coupled with substantial pesticide application, large quantities of NEOs are introduced into water bodies through atmospheric deposition and surface runoff, resulting in downstream migration of contamination to urban areas [25]. Consequently, residents of PRD cities with high water utilization—such as Guangzhou, Shenzhen, and Foshan—are at increased risk of NEO pollution. Environmental monitoring has also revealed that residual NEO concentrations in the Pearl River rank among the highest in China, corroborating that urban residents in this region—particularly children and other vulnerable populations—are at considerable exposure risk [26]. These regional characteristics provide an ideal context for assessing the health impacts of chronic, low-level, mixed NEO exposure under real-world environmental conditions.

Previous research indicates that urinary neonicotinoids and their metabolites can serve as biomarkers of total exposure from multiple sources based on human metabolic characteristics [27]. Therefore, this study aimed to assess the associations between real-life mixed exposures to NEOs and their urinary metabolites and neurobehavioral development in preschool children living in Shenzhen in the Pearl River Delta of China. Our findings are expected to provide epidemiological evidence and scientific insights to support the identification of environmental health risks during critical windows of childhood neurodevelopment and inform future policy interventions at the national and global levels.

## 2. Materials and Methods

### 2.1. Research Design and Study Participants

**Figure 1:**
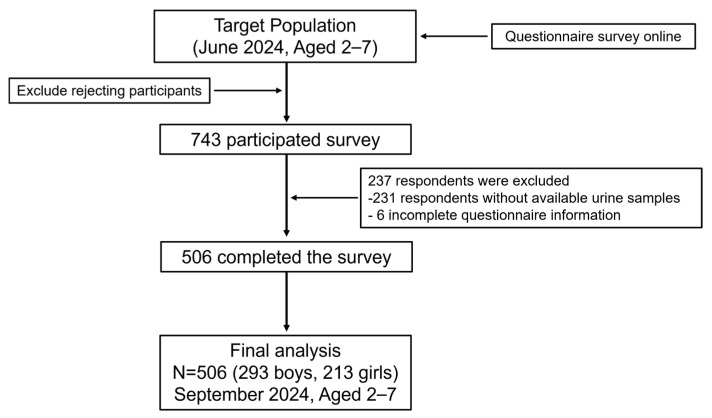
Flowchart of population included in final analysis (*n* = 506).

This cross-sectional study was conducted in Shenzhen, Guangdong Province, from June to September 2024, using a random cluster sampling approach to recruit participants. Shenzhen consists of ten administrative districts. In this study, one district was randomly selected from the list of all districts using a random number generator, and Bao’an District was determined as the study site. The expected sample size was proportionally allocated across subdistricts within Bao’an District according to the number of preschool-aged children. Finally, one to two kindergartens were randomly selected from each subdistrict using a random number table, and children attending these kindergartens were invited to participate in the study. The inclusion criteria were (1) residence within the study area and (2) no diagnosis of major diseases (e.g., severe cardiovascular conditions or psychiatric disorders). Exclusion criteria included (1) residence outside the study area or recent migration into the area within the past year; (2) presence of major illnesses as described above, including diagnosed neurodevelopmental or psychiatric disorders such as Autism Spectrum Disorder (ASD), attention-deficit/hyperactivity disorder (ADHD), or other clinically recognized behavioral abnormalities; and (3) failure to complete all study questionnaires. A total of 743 questionnaires were collected, and 512 participants also provided urine samples. After data validation, 6 participants were excluded due to missing key questionnaire information. The final sample included 506 preschool-aged children who met both the inclusion and exclusion criteria and were included in subsequent analyses. The study design and participant selection process are illustrated in Figure 1.

All participants (with written consent provided by legal guardians for those under 18 years of age) gave informed consent prior to participation. The study protocol was approved by the Ethics Committee of the School of Public Health, Sun Yat-sen University (Approval No. IRB-SYSU-2024-104).

### 2.2. Analysis of Neonicotinoids in Urine

#### 2.2.1. Sample Preparation

According to the preprocessing and computer application methods reported in relevant literature, the urine samples were processed according to the following operation steps after the optimization of experimental conditions [28,29,30]. One night in advance, transfer the urine sample from the −80 refrigerator to the 4 °C refrigerator for dissolution, vortex for 20 s, transfer 1 mL of urine to a 15 mL glass tube, add 0.3 mL of ammonium acetate buffer solution (prepared as 7.7 g ammonium acetate, 6 mL glacial acetic acid, 100 μL β—glucuronidase, and adding water for the volume to reach 100 mL), add 20 μL of 100 ppb internal standard mixture, and incubate at 37 °C for 6 h. Add 0.5 mL of acetonitrile, vortex for 10 s and mix well. Add 7 mL ethyl acetate, sonicate for 30 min, shake for 30 min, centrifuge at 3400× *g* for 5 min, and take the supernatant (repeat twice). Add 10 mL n-hexane, sonicate for 30 min, shake for 30 min, and centrifuge at 3400× *g* for 5 min. Combine the supernatant, blow dry with nitrogen, add 1 mL n-hexane, shake for 5 min, blow dry with nitrogen, then redissolve with 200 μL methanol water, filter through a 0.22 μm organic filter membrane, and finally transfer to the injection vial for sample injection. A pure water blank with pretreatment steps completely consistent with the above operations is set for every 20 samples.

#### 2.2.2. HPLC-MS/MS Analysis and Ion Source Optimization

HPLC-MS/MS was a Xevo TQ-XS (Milford, MA, USA) from Waters. The chromatographic column was a BEH C18 column (2.1 mm × 100 mm, particle size 5 μm) from Waters. After multiple experiments, a mobile phase composed of 0.1% Fa + water (A) and methanol (B) was selected as optimum. The initial mobile phase ratio was 95% A, then changed to 100% B within 4 min, and then continued for 5 min, then changed to 95% A within 1.1 min and maintained for 3.9 min. Mass spectrometry analysis was performed on a mass spectrometry detector analyzer equipped with an ESI source. The ion source temperature was set at 400 °C. The data were processed and exported using Waters software masslynx4.2. See Table A1 for specific mass spectrometric parameter information of each substance.

#### 2.2.3. Urinary Creatinine Assay

Urinary creatinine concentration was determined using a creatinine assay kit (Nanjing Jincheng Bioengineering Inst., Nanjing, China) based on the sarcosine oxidase method. Creatinine is converted to creatine by creatinine; creatine is then converted to sarcosine and urea by creatines; sarcosine is subsequently oxidized by sarcosine oxidase to generate hydrogen peroxide, which reacts with a chromogenic reagent to form a colored product. Absorbance was measured at 546 nm. For the analysis, urine samples were diluted 1:10 with deionized water, and creatinine concentrations were calculated using the formula provided in the kit instructions.

### 2.3. Assessment of Neurobehavioral Problems in Preschool Children

Neurobehavioral problems in preschool-aged children were assessed using the Strengths and Difficulties Questionnaire (SDQ). The SDQ is a parent-reported instrument comprising 25 items divided into five subscales: emotional symptoms, conduct problems, hyperactivity, peer relationship problems, and prosocial behavior. Each subscale contains five items, and responses are scored on a three-point Likert scale (0 = not true, 1 = somewhat true, 2 = certainly true), yielding subscale scores ranging from 0 to 10. Higher scores on the first four subscales indicate more severe symptoms, while higher scores on the prosocial behavior subscale indicate more positive behavior. In addition to subscale scores, a total difficulties score was calculated by summing the scores of the four problem-oriented subscales (emotional symptoms, conduct problems, hyperactivity/inattention, and peer problems), providing a composite index of children’s overall behavioral and emotional difficulties [31]. Based on established cutoffs in the previous literature, threshold values of 5, 4, 7, 4, and 17 were used for emotional symptoms, conduct problems, hyperactivity, peer problems, and total difficulties, respectively [32,33]. Due to its brevity (25 items), the SDQ is generally well accepted by parents and is suitable for use in large-scale epidemiological studies. The validity and reliability of the SDQ have been well established in diverse populations [34]. In the present study, the Cronbach’s alpha coefficients indicated acceptable internal consistency (α = 0.769), supporting the instrument’s reliability for assessing neurobehavioral outcomes in this population.

### 2.4. Statistical Analysis

Continuous variables were summarized using means and standard deviations or medians and interquartile ranges, as appropriate. Categorical variables were described using counts and percentages. To account for differences in the toxicological potency of individual NEOs on human health and to facilitate comparability across studies, we applied the Relative Potency Factor (RPF) approach to normalize each NEO to an imidacloprid equivalent (IMIeq), denoted as IMIRPF. The reference doses (RfDs) used for this calculation were derived from the 2021 Human Health Benchmarks for Pesticides, published by the U.S. Environmental Protection Agency (USEPA) (available at: https://www.epa.gov/sdwa/2021-human-health-benchmarks-pesticides (accessed on 15 January 2025) 2021 Human Health Benchmarks for Pesticides—US EPA), which reflect the relative human health toxicity of each compound (Table A2) [35,36]. For neonicotinoids without available EPA cRfD data, RPFs were estimated based on structurally similar compounds following previously reported approaches [37]. RPFs were calculated using the following formula: RPF_i_ = RfD_IMI_/RfD_i_, where i denotes an individual NEO. Accordingly, the imidacloprid-equivalent concentration (IMIeq) for each NEO was calculated by multiplying its measured concentration by the corresponding RPF_i_. The final formula for IMIeq (ng/mL) was as follows:

IMI_eq_ (ng/mL) (NEO_i_×RPF_i_) = IMI + 0.82 × CLO + 6.67 × THM + 0.08 × DNT + 0.11 × NIT + 1.6 × SFX + 1.13 × NACE + 6.67 × THM + 1.13 × ACE + 20 × THD.

**Figure A1:**
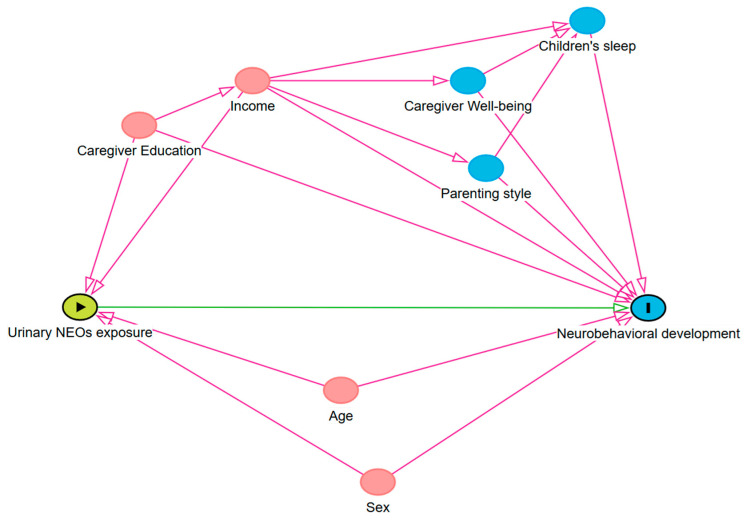
Directed acyclic graph (DAG) used to select potential covariates in the associations between urinary NEO exposure and measures of neurobehavioral development in preschool children.

Concentrations below the limit of quantification (LOQ) were substituted with LOQ divided by the square root of 2 (LOQ/√2) for statistical analysis. All subsequent statistical analyses were conducted using creatinine-adjusted urinary concentrations (μg/g Cr) to account for urine dilution. Prior to statistical analysis, the concentrations of neonicotinoid insecticides were log-transformed. A Directed Acyclic Graph (DAG) was constructed to select covariates (Figure A1), based on existing literature and expert knowledge [38,39]. The selected covariates included sex (boy, girl), child’s age (continuous, years), educational level of the primary caregiver (primary school or below, junior high school, senior high school, college or above), and monthly household income (<2500 CNY, 2500–7500 CNY, 7500–15,000 CNY, >15,000 CNY). Parenting style of the primary caregiver and sleep conditions of the child were also included as confounding factors due to their influences on neurobehavioral development [40,41].

Multiple linear regression was first used to examine the association between neonicotinoid insecticides and neurobehavioral development. Based on the outcome dimensions that showed potential associations in the regression results, binary logistic regression was further conducted to estimate odds ratios (ORs) and 95% confidence intervals (CIs) for abnormal versus normal neurobehavioral outcomes. Given evidence from previous literature suggesting that neonicotinoid insecticides may have sex-specific effects, stratified analyses by sex were also performed.

The Weighted Quantile Sum (WQS) regression model was used to assess the effect of mixed exposure to neonicotinoid compounds. The WQS model constructs a weighted index by combining the concentrations of multiple chemicals and assigning weights based on the strength of their associations with the health outcome, thereby estimating the overall effect of the mixture. Specifically, WQS regression transforms the concentrations of each neonicotinoid into quartiles to build the index and assigns weights according to their associations with neurobehavioral development to evaluate the joint exposure effects [42].

The Bayesian Kernel Machine Regression (BKMR) model was further applied to assess the effects of mixed exposure to neonicotinoid compounds. BKMR is a flexible Bayesian method capable of accounting for nonlinear and interactive effects among exposure variables. Unlike WQS regression, BKMR estimates the overall effect of multiple exposures on health outcomes by simulating changes in the mixture and modeling the exposure–response relationship through 10,000 iterations. The model provides posterior inclusion probabilities (PIPs) for each compound, with a PIP > 0.50 indicating a significant contribution to the outcome. This approach allows for the identification of key contributors within the exposure mixture and the evaluation of both independent and interactive effects [43].

## 3. Results

### 3.1. SDQ Scores by Demographic Characteristics

This study included 506 preschool children, aged from 2 to 7 years. Of these, 293 were boys (57.9%) and 213 were girls (42.1%). In terms of household characteristics, the largest proportion of families (33.6%) reported a monthly income exceeding 15,000 CNY, and most primary caregivers had completed high school education (66.2%). The mean scores for the SDQ subscales were as follows: emotional symptoms, 1.74 (M = 1.78); conduct problems, 1.86 (M = 1.28); hyperactivity, 3.70 (M = 2.19); peer problems, 2.33 (M = 1.54); and total difficulties, 9.63 (M = 4.82). Participant characteristics are presented in Table 1.

### 3.2. Urinary Concentrations of Neonicotinoids

Urinary concentrations and detection rates of 11 neonicotinoid compounds among the 506 preschool children are presented in Table 2. High detection rates were noted for imidacloprid (IMI, 97.4%), clothianidin (CLO, 100%), thiamethoxam (THM, 100%), dinotefuran (DNT, 99.8%), nitenpyram (NIT, 99.8%), nitenpyram-N-desmethyl (NACE, 100%), and thiacloprid-N-desmethyl (NTHM, 99.8%). The mean concentrations of these compounds were as follows: 0.452 μg/g Cr (IMI), 14.165 μg/g Cr (CLO), 10.471 μg/g Cr (THM), 23.925 μg/g Cr (DNT), 7.767 μg/g Cr (NIT), 5.456 μg/g Cr (NACE), and 21.328 μg/g Cr (NTHM). The detection rates for flonicamid (FLO, 82.2%), sulfoxaflor (SFX, 69.4%), and thiacloprid (THD, 67.6%) were comparatively lower, with mean concentrations of 0.069 μg/g Cr, 0.382 μg/g Cr, and 0.018 μg/g Cr, respectively. However, 6-chloronicotinic acid (6-CINA) showed a low detection rate (6.10%), leading to the exclusion of its concentration data from subsequent analyses to maintain analytical validity and reliability. The overall mean concentration of neonicotinoid compounds in the study population was 234.145 μg/g Cr.

### 3.3. Association Between Neonicotinoid Concentrations and Neurobehavioral Problems, and the Effect Modification by Gender

#### 3.3.1. Generalized Linear Regression Model

**Figure 2:**
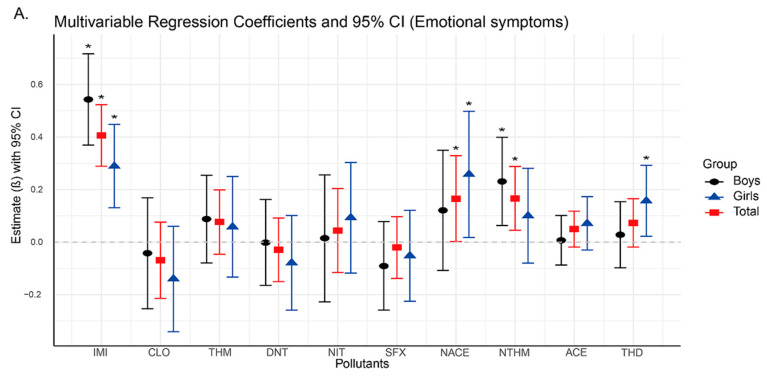

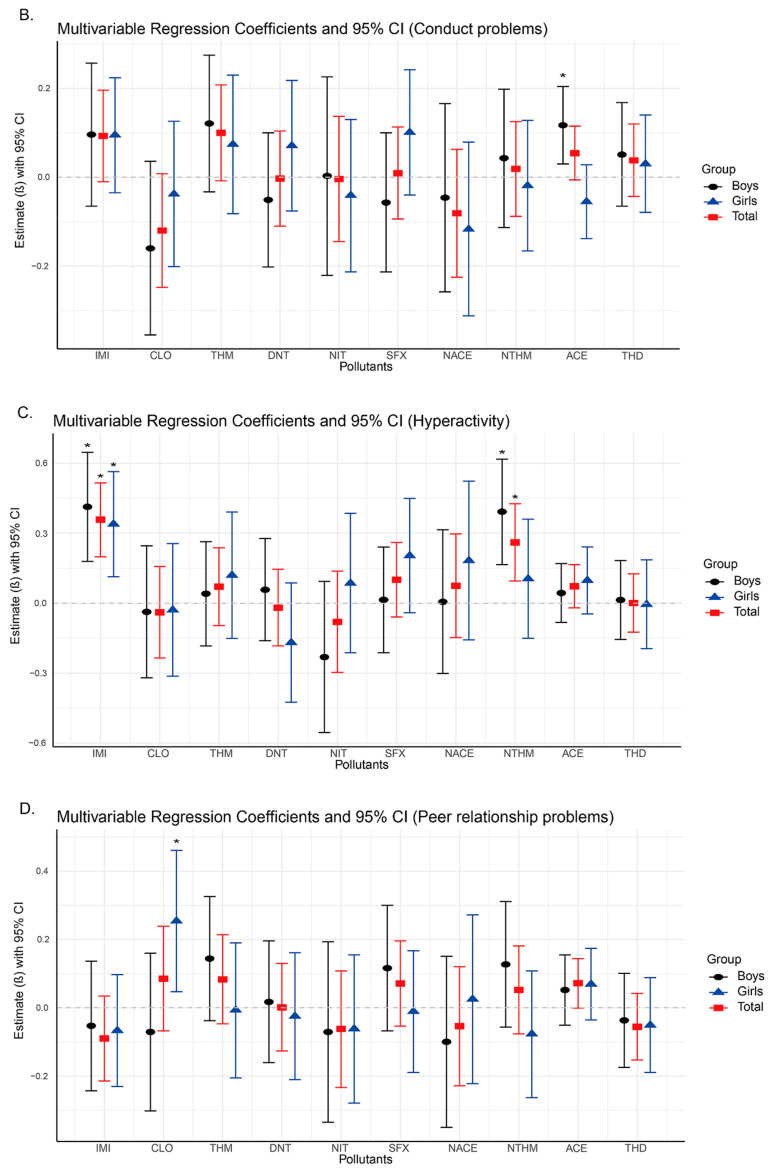

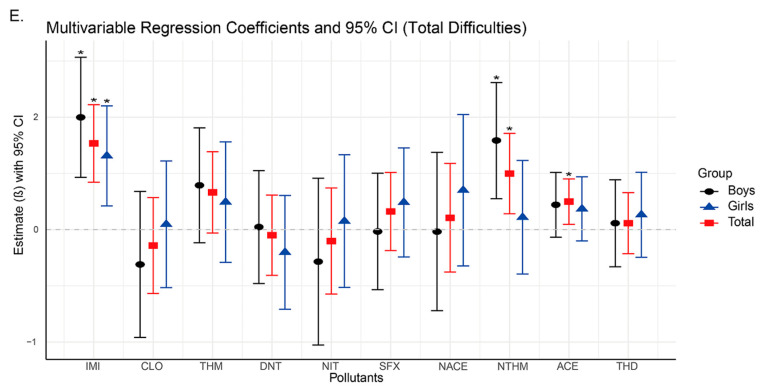
Changes in neurobehavioral symptoms associated with neonicotinoid use based on linear regression models (95% confidence intervals). Asterisks indicate significant differences with *p* < 0.05.

In this study, we first used multiple linear regression models to evaluate the associations between urinary concentrations of NEOs and neurobehavioral issues in preschool children, including emotional symptoms, conduct problems, hyperactivity, peer relationship problems, and total difficulties. The regression results are illustrated in Figure 2.

In our study, the analysis results showed that significant positive associations were found between emotional symptoms and the urinary concentrations of IMI (β = 0.406, 95% CI: 0.289, 0.523), NACE (β = 0.165, 95% CI: 0.002, 0.329), and NTHM (β = 0.406, 95% CI: 0.045, 0.288) in the total population (Figure 2A,C,E), indicating that higher exposure levels were associated with a greater likelihood of emotional problems. In terms of hyperactivity, significant positive associations were observed for both IMI (β = 0.358, 95% CI: 0.199, 0.516) and NTHM (β = 0.261, 95% CI: 0.096, 0.426). Regarding total difficulties, which is a composite measure derived from the four problem subscales, significant associations were identified for IMI (β = 0.767, 95% CI: 0.422, 1.111), NTHM (β = 0.498, 95% CI: 0.141, 0.856), and ACE (β = 0.249, 95% CI: 0.047, 0.451). No significant associations were observed for conduct problems or peer relationship problems in the total sample (Figure 2B,D).

Sex-stratified analyses revealed differences in the associations of NACE and NTHM with neurobehavioral outcomes. For emotional symptoms (Figure 2A), NACE was significantly associated with the total population and girls (β = 0.258, 95% CI: 0.018, 0.498), but not with boys. In contrast, NTHM showed significant positive associations with the total population and boys (β = 0.231, 95% CI: 0.063, 0.399), but not with girls. Similarly, for hyperactivity and total difficulties, NTHM was significantly associated with both the total population and boys (hyperactivity: β = 0.392, 95% CI: 0.166, 0.618; total difficulties: β = 0.793, 95% CI: 0.276, 1.309), with no significant associations found in girls.

To further explore the impact of neonicotinoid exposure on neurobehavioral problems, we constructed binary logistic regression models for outcome dimensions that demonstrated potential associations in the multiple linear regression: emotional symptoms, conduct problems, and total difficulties. We calculated odds ratios (ORs) and 95% confidence intervals (CIs) to estimate the association between each neonicotinoid compound and neurobehavioral abnormalities (Table 3, Table 4 and Table 5). The results indicated that IMI exhibited significant positive associations across all three dimensions in the total population: emotional symptoms (OR = 2.194, 95% CI: 1.490, 3.229), hyperactivity (OR = 1.692, 95% CI: 1.197, 2.392), and total difficulties (OR = 1.616, 95% CI: 1.124, 2.323). This result suggests that increased IMI exposure may elevate the risk of neurobehavioral problems. In sex-stratified analyses, significant positive associations were observed in both boys and girls for emotional symptoms and hyperactivity. For boys, the odds ratios were as follows: emotional symptoms (OR = 2.743, 95% CI: 1.532, 4.912); hyperactivity (OR = 1.834, 95% CI: 1.139, 2.954). For girls, the odds ratios were as follows: emotional symptoms (OR = 2.727, 95% CI: 1.200, 6.200); hyperactivity (OR = 1.961, 95% CI: 1.070, 3.593). However, a significant positive association for total difficulties was observed only among girls (OR = 2.223, 95% CI: 1.101, 4.488). Furthermore, after sex stratification, the results for NACE and NTHM revealed distinct sex-specific differences. Among girls, NACE exhibited significant positive associations across all three dimensions: emotional symptoms (OR = 5.692, 95% CI: 1.910, 16.958), hyperactivity (OR = 2.567, 95% CI: 1.189, 5.542), and total difficulties (OR = 3.512, 95% CI: 1.402, 8.796). No significant associations were observed for NACE in boys. In contrast, NTHM was significantly associated with hyperactivity (OR = 1.513, 95% CI: 1.042, 2.196) and total difficulties (OR = 1.605, 95% CI: 1.078, 2.390) exclusively in boys, with no corresponding associations identified in girls. These findings further support the potential sex-specific heterogeneity in the neurobehavioral effects of NACE and NTHM exposure.

#### 3.3.2. WQS Regression Model

**Figure 3:**
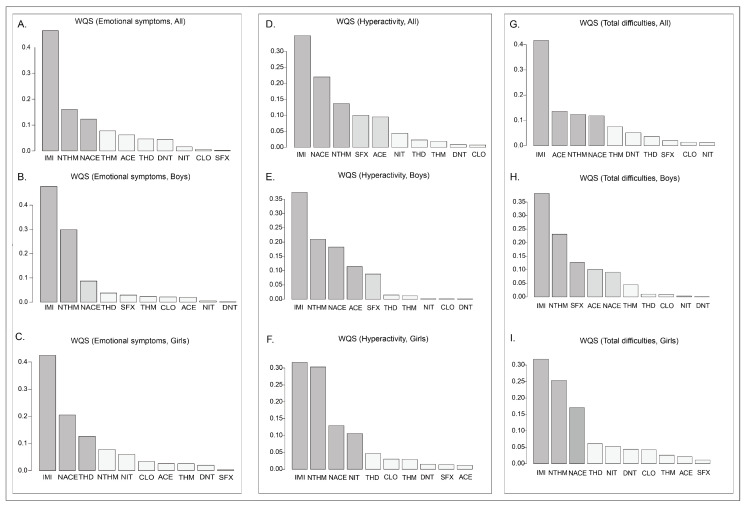
WQS regression weights between the concentrations of neonicotinoid substances in the urine of preschool children and various dimensions of neurobehavior.

To further investigate the combined effects of neonicotinoid compounds on neurobehavioral problems in preschool children, Weighted Quantile Sum (WQS) regression models were applied to assess their joint impact. Based on the outcome dimensions that demonstrated significant associations in prior regression analyses, WQS models were constructed for emotional symptoms, hyperactivity, and total difficulties, followed by sex-stratified analyses. After adjusting for all covariates in the total population, the WQS index showed statistical significance for emotional symptoms (*p* < 0.001), hyperactivity (*p* < 0.001), and total difficulties (*p* < 0.001) (Table A3). In the emotional symptoms model, the primary contributors with weights exceeding 0.1 were IMI (0.464), NTHM (0.160), and NACE (0.123) (Figure 3A, Table A4). In the hyperactivity model, the main contributors included IMI (0.349), NACE (0.220), and NTHM (0.136) (Figure 3D, Table A5). In the total difficulties model, the primary contributors included IMI (0.416), ACE (0.136), NTHM (0.124), and NACE (0.118) (Figure 3G, Table A6).

In addition, we conducted sex-stratified WQS regression models. The WQS indices displayed statistically significant positive effects in both boys and girls (*p* < 0.001). For boys, the primary contributors (weights > 0.1) in the emotional symptoms model were IMI (weight = 0.478) and NTHM (0.298); the main contributors in the hyperactivity model included IMI (0.374), NTHM (0.210), NACE (0.183), and ACE (0.114) (Figure 3B,E, Table A5). The major contributors in the model of total difficulties were IMI (0.382), NTHM (0.232), SFX (0.127), and ACE (0.102) (Figure 3H, Table A6). For girls, the key contributors to emotional symptoms were IMI (0.424), NACE (0.205), and THD (0.127) (Figure 3C, Table A4); the major contributors in the hyperactivity model included IMI (0.316), NTHM (0.303), NACE (0.130), and NIT (0.106) (Figure 3F, Table A5); and the primary contributors in the model of total difficulties were IMI (0.318), NTHM (0.253), and NACE (0.170) (Figure 3I, Table A6).

In summary, the results of the WQS model demonstrated significant combined effects of NEOs exposure on emotional symptoms, hyperactivity, and total difficulties, mainly driven by IMI, NTHM, and NACE.

#### 3.3.3. BKMR Model

**Figure 4:**
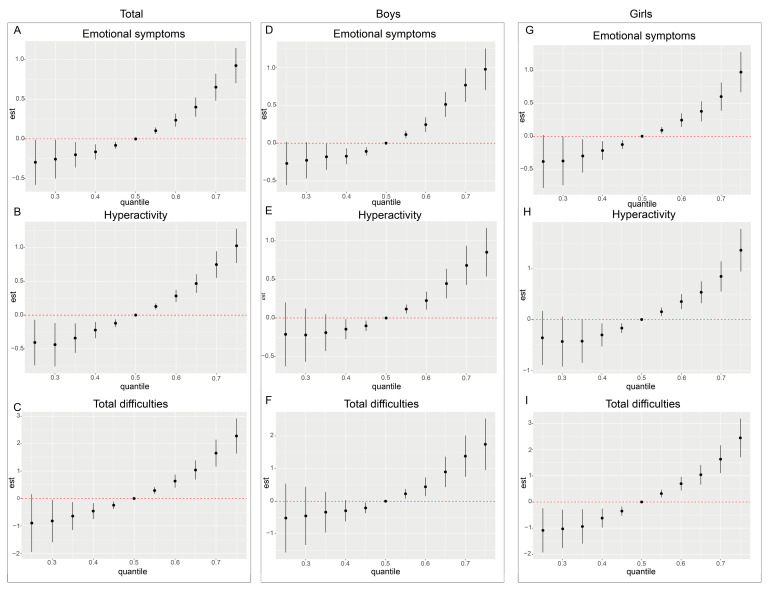
Cumulative effects (95% CI) of the neonicotinoid mixture on the emotional problems dimension (**A**–**C**), the hyperactivity problems dimension (**D**–**F**), and the total difficulties dimension (**G**–**I**) for the whole population and for boys versus girls when all specific percentiles of the analytes were compared to all 50th percentiles of the analytes.

Due to the limitations of the Weighted Quantile Sum (WQS) regression model in accounting for nonlinear relationships among exposure variables, we further applied Bayesian Kernel Machine Regression (BKMR) to assess the nonlinear joint effects of exposure to ten NEOs on neurobehavioral issues in preschool children. The BKMR model concentrated on three SDQ dimensions (emotional symptoms, hyperactivity, and total difficulties), which were positively correlated with the exposure to NEOs in the aforementioned models. To investigate potential effect modification by gender, sex-stratified analyses were conducted. After adjusting for all covariates, the BKMR model estimated the overall mixture effect of the ten neonicotinoid compounds (Figure 4).

The results indicated that when the concentrations of all compounds exceeded the median exposure level (50th percentile), the estimated values for emotional symptoms, hyperactivity, and total difficulties exhibited a monotonic increasing trend. This trend was statistically significant at multiple percentile points, suggesting that exposure to NEOs may have a cumulative adverse effect on neurobehavioral outcomes. Additionally, sex-stratified analyses revealed that the positive joint effects of NEO mixture exposure were evident in both boys and girls, with no significant sex-specific differences observed. These findings indicate a consistent association between NEO exposure and neurobehavioral outcomes across sexes. In all three outcome dimensions and across sex-stratified subgroups, the estimated values of neurobehavioral problems were lower when all compound concentrations were below the 50th percentile. Notably, within the 40th to 50th percentile range, several subgroups displayed statistically significant differences compared to the 50th percentile.

**Figure 5:**
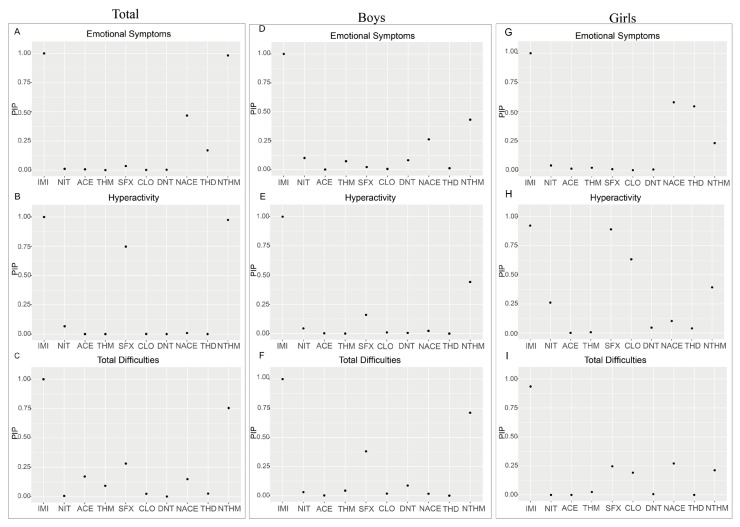
PIP analysis of different neonicotinoids on emotional symptoms (**A**–**C**), hyperactivity (**D**–**F**), and total difficulties (**G**–**I**) dimensions among the total preschool population, boys, and girls using the BKMR model.

In subsequent analyses, the Posterior Inclusion Probability (PIP) was used to evaluate the relative importance of each exposure variable in the model (Figure 5). IMI, NTHM, and NACE exhibited higher PIP values in the emotional symptoms and total difficulties models. Conversely, compounds such as THD and ACE demonstrated relatively lower PIP values across multiple dimensions.

## 4. Discussion

This study is an epidemiological investigation to systematically assess urinary concentrations of NEOs and their associations with neurobehavioral development in a preschool-age population. Among the 11 neonicotinoid substances and their metabolites measured, five neonicotinoid prototypes, including IMI, CLO, THM, DNT, and FLO, and two of their metabolites (NACE and NTHM) were detected in over 90% of samples. The mean total urinary concentration of NEOs reached 234.145 μg/g Cr, which exceeds the exposure levels reported in previous studies, suggesting that preschool children in China may be facing substantial exposure risks to neonicotinoids [44]. The detection rates observed in this study are consistent with the results reported from recent population studies in China, in which most of the neonicotinoids tested showed high detection frequencies [26]. However, in earlier population studies, the reported detection rates were generally lower [45,46]. In addition, the overall human exposure to neonicotinoids and the detection rates of individual compounds reported in the literature have shown an upward trend over time. For instance, a 2020 epidemiological survey among children in Shanghai reported detection rates ranging from 0.6% to 62.6% [46], whereas a 2016 survey conducted in Japan reported detection rates of ≤57.8%. This upward trend in detection metrics suggests that preschool children and the general population may now be facing higher exposure risks [45].

Notably, this study observed considerable inter-individual differences in urinary neonicotinoid concentrations. Such variations may reflect disparities in the recent living environments of the preschool participants, including the use of household insecticides and the presence of pesticide residues in fruits, vegetables, and drinking water. Only a very small proportion of participants exhibited extremely high concentrations, which are likely to represent short-term, episodic exposure events rather than chronic accumulation. Nevertheless, these abnormally elevated levels merit future attention, as such episodic exposures may pose potential health risks to sensitive populations, particularly preschool children.

Subsequent regression analyses revealed significant positive associations between urinary concentrations of IMI, NTHM, and NACE and neurobehavioral problems in preschoolers, particularly in the domains of emotional symptoms, hyperactivity, and total difficulties. As the concentrations of IMI, NTHM, and NACE increased, the risk of neurobehavioral abnormalities also appeared to rise. These compounds may exert adverse neurodevelopmental effects by activating nicotinic acetylcholine receptors (nAChRs) in the nervous system [21]. Early studies suggested that the neurotoxicity of neonicotinoids was low because mammals, especially humans, have relatively few nAChRs in the brain with low binding affinity [47]. However, more recent animal and mechanistic studies have challenged this view [24]. Several commonly used insecticides, including neonicotinoids, have demonstrated neurotoxic effects on the developing nervous system to varying degrees. IMI has been shown in brainstem slices from neonatal mice to induce depolarizing inward currents, increase neuronal excitability, and provide direct physiological evidence of neurotoxicity [21]. In a mouse study, low-dose exposure to neonicotinoids such as sulfoxaflor impaired performance in elevated plus maze tests and motor coordination in juvenile mice [48]. Mechanistic data suggested that nAChRs and other mediators of neurodevelopment play crucial roles during fetal and early postnatal brain development, with particularly high expression during infancy [49]. Exposure to certain neonicotinoids during this developmental window may disrupt nAChR expression and regulation, interfere with neuronal migration and synaptogenesis, and affect other developmental processes, potentially yielding adverse neurobehavioral outcomes [49]. Collectively, the above evidence indicates that neonicotinoid insecticides or their metabolites can act on the nervous system of mammals, including humans, altering neuronal signaling and inducing toxic or oxidative stress–related effects in developing neurons. Therefore, the neurotoxic potential of neonicotinoids in mammals may have been underestimated, and continued scientific and regulatory attention to their safety is warranted.

The findings of this study suggest that among the detected neonicotinoid insecticides, IMI, NTHM, and NACE are the most influential compounds associated with neurobehavioral outcomes in preschoolers. Notably, IMI showed consistent and statistically significant positive associations across all strata (overall population, boys, and girls). Together with the WQS regression results, IMI emerged as a key contributor within the mixture. To date, no epidemiological studies have directly examined IMI exposure and neurobehavioral outcomes in humans; however, experimental evidence suggests that IMI’s potent effects may arise from interference with neurotransmitter systems and the induction of oxidative stress [50]. For instance, in a prior study using cultured cerebellar neurons from neonatal rats, IMI significantly enhanced the activity of nAChR subunits α3, α4, and α7, an effect not observed with other neonicotinoids [21]. In a mouse low-dose IMI exposure reduced SOX2-positive neural stem cells and GFAP-positive astrocytes, indicating suppressed neurogenesis and glial development in the dentate gyrus (DG) [51]. Behavioral impairments were observed in the elevated plus maze (EPM) and fear conditioning (FC) tests, suggesting anxiety-like behavior and deficits in learning and memory [48]. Collectively, these mechanistic studies suggest that IMI may exert a more pronounced neurotoxic impact than other neonicotinoids and threaten neurobehavioral development in preschool-aged children during early life. Therefore, given the consistently observed adverse effects of IMI across different subgroups, targeted mitigation strategies addressing IMI exposure may reduce health risks from neonicotinoids in young children and the general population.

In this study, both NTHM (a metabolite of THM) and NACE (a metabolite of ACE) showed significant positive associations with neurobehavioral outcomes, whereas the parent compounds (THM and ACE) showed no such associations. Prior toxicokinetic studies have shown that demethylated metabolites such as NTHM and NACE are more hydrophilic and possess slower metabolic clearance and longer biological half-lives, resulting in more persistent internal exposure and potentially stronger biological effects than their parent compounds [52]. For example, a 2022 study reported that NACE could be consistently detected in the cerebrospinal fluid (CSF) of children, whereas its parent compound was less frequently detected, and that CSF NACE concentrations correlated linearly with those in blood and urine. These findings suggest that these NEO metabolites can remain in the human body for a longer time and penetrate the central nervous system [53]. Mechanistic studies further revealed that demethylated THM has 28–3600 times higher receptor affinity than THM, and THM itself resembles a pro-pesticide, with its neuroactivity primarily mediated by its transformation products [52].

In addition, NTHM and NACE show notable sex-specific differences in stratified analyses. Among boys, NTHM is positively associated with hyperactivity and total difficulty scores, whereas no such associations are observed among girls. These findings may partly reflect sex-related differences in the metabolism of neonicotinoids [54,55]. However, the mechanisms underlying the observed sex differences in NACE and NTHM effects remain poorly understood and require further investigation to elucidate how these compounds may differentially influence neurobehavioral development in boys and girls through distinct physiological pathways.

Subsequent BKMR analysis shows that urinary neonicotinoid concentrations are positively associated with neurobehavioral problem scores across all population subgroups, with prominent effects on emotional symptoms, hyperactivity, and overall difficulties. Notably, effect estimates rose with exposure and remained statistically significant across all percentile levels. These results suggest that even low levels of neonicotinoid exposure may have adverse effects on the neurobehavioral development of preschool children. This finding aligns with prior in vivo and in vitro studies showing that low-dose neonicotinoid exposure can induce nicotine-like neurotoxicity [56]. Additional experimental work indicates that neonicotinoids, even at environmentally relevant concentrations, can activate nAChRs, induce oxidative stress, and damage synaptic structure, thereby disrupting neuroplasticity and regulatory function in the developing nervous system [57]. Taken together, IMI, NTHM, and NACE showed significant positive associations in the regression models, whereas the contributions of other neonicotinoids were relatively limited. This suggests that particular attention should be paid to the potential health risks of IMI, THM (the parent compound of NTHM), and ACE (the parent compound of NACE). From a public health perspective, reducing the use of these compounds and exploring safer alternatives may help mitigate their potential adverse effects on neurobehavioral development in preschool children.

Nevertheless, this study has several limitations that warrant acknowledgment. First, because this is a cross-sectional study, it cannot establish causal relationships between neonicotinoid exposure and neurobehavioral outcomes. Second, most neonicotinoids have relatively short biological half-lives in humans, and the spot urine samples collected in this study may not accurately reflect long-term cumulative exposure. In addition, because urine collection did not occur immediately after potential exposure events, transient peak concentrations may have been missed, meaning that the actual short-term exposure levels could have been underestimated. Meanwhile, the total IMIeq concentrations calculated using RPFs based on EPA reference doses primarily serve to reflect overall exposure levels for comparability across studies, rather than to precisely represent differences in neurotoxic potency, as the underlying RfDs were derived from heterogeneous toxicological endpoints. Third, neurodevelopmental outcomes were assessed with the SDQ, which may be affected by recall bias and other reporting inaccuracies. Finally, the sample size of this study was relatively modest (*n* = 506), and all participants were recruited from a single city (Shenzhen), which may limit generalizability and robustness of the findings. Future prospective cohort studies and experimental investigations are warranted to provide more comprehensive evidence on the impact of neonicotinoid exposure on human neurobehavioral development.

## 5. Conclusions

The findings suggest that exposure to neonicotinoid insecticides may be associated with neurobehavioral problems in preschool-aged children. Among the compounds examined, IMI, NTHM, and NACE emerged as the primary contributors and showed significant positive associations with neurobehavioral problems. In addition, NTHM and NACE exhibited sex-specific differences in this population. The BKMR analyses revealed that the combined effects of neonicotinoid mixtures were also positively associated with neurobehavioral difficulties, indicating potential adverse impacts even at low exposure levels. Therefore, future efforts should prioritize reducing the use of IMI, THM, and ACE in agricultural practices and using safer alternatives to mitigate their potential risks to children’s neurodevelopment. Future studies should involve larger sample sizes and complementary in vitro and in vivo experiments to validate and extend these findings, thereby informing early identification of environmental health risks and preventive strategies for vulnerable pediatric populations.

## Figures and Tables

**Table 1:** Sociodemographic, psychological, and behavioral characteristics of participants.

Characteristics	*n* (%)	Mean (SD)
**Children’ s characteristics**		
Age (years)		5.46 (0.95)
Gender		
Boys	293 (57.9)	
Girls	213 (42.1)	
**Children’ s emotional and behavioral problems**		
SDQ emotional symptoms score		1.74 (1.78)
SDQ conduct problems score		1.86 (1.28)
SDQ hyperactivity score		3.70 (2.19)
SDQ peer problems score		2.33 (1.54)
SDQ total difficulties score		9.63 (4.82)
**Primary caregivers’ characteristics**		
Economic situation (per capita)		
<2500 CNY ^a^	51 (10.1)	
2500–7500 CNY	146 (28.9)	
7500–15,000 CNY	139 (27.5)	
>15,000 CNY	170 (33.6)	
Education level of the primary caregiver		
Primary school or below	34 (6.70)	
Junior high school	98 (19.4)	
High school	335 (66.2)	
University and above	19 (3.75)	

^a^ CNY = ChineseYuan.

**Table 2:** Concentrations of neonicotinoids in the urine of preschool children (*n* = 506).

	LOD (ng/mL)	Detection Rate (%)	Unadjusted (ng/mL)				Creatinine Adjusted (μg/g Cr)			
			Range	Percentile			Range	Percentile		
				25th	50th	75th		25th	50th	75th
IMI	0.0044	97.4%	<LOD–3.913	0.034	0.071	0.165	<LOD ^a^–32.745	0.037	0.090	0.258
CLO	0.0024	100%	0.047–119.284	1.196	2.417	4.382	0.024–1705.401	1.378	3.023	7.242
THM	0.0042	100%	0.039–307.508	0.533	1.168	2.638	0.035–1119.698	0.625	1.468	3.801
DNT	0.0081	99.8%	<LOD–521.604	2.400	5.091	10.590	<LOD ^a^–773.853	3.026	6.217	16.269
NIT	0.0241	99.8%	<LOD–107.599	1.089	1.806	3.034	<LOD ^a^–824.314	1.214	2.319	5.169
SFX	0.0579	69.4%	<LOD–7.397	<LOD	0.099	0.219	<LOD ^a^–8.152	<LOD	0.132	0.345
ACE	0.0006	63.5%	<LOD–1.001	<LOD	0.005	0.015	<LOD ^a^–2.994	<LOD	0.006	0.024
THD	0.0014	67.6%	<LOD–0.128	<LOD	0.004	0.011	<LOD ^a^–0.732	<LOD	0.005	0.016
FLO	0.0005	82.2%	<LOD–1.360	0.001	0.008	0.027	<LOD ^a^–3.218	0.002	0.010	0.038
6-CINA	0.0070	6.10%	<LOD–0.089	<LOD	<LOD	<LOD	<LOD ^a^–0.123	<LOD	<LOD	<LOD
NACE	0.0006	100%	0.145–20.245	0.816	1.350	2.551	0.058–236.500	0.881	1.886	4.422
NTHM	0.0350	99.8%	<LOD–295.988	0.578	1.118	2.114	<LOD ^a^–6006.487	0.717	1.445	3.106
Σ_10_NEOs			3.003–2114.537	14.571	23.803	45.249	1.638–45,357.346	17.135	31.481	68.591

^a^ Below the limits of detection for the urinary concentrations were not corrected for creatinine.

**Table 3:** Odds ratios (ORs) and 95% confidence intervals (CIs) of urinary NEO concentrations and emotional problem dimensions in preschool children.

NEOs	Binary Logistic Regression OR (Emotional Problem, 95% CI)		
	Total	Boys	Girls
IMI	2.194 (1.490, 3.229) ***	2.743 (1.532, 4.912) ***	2.727 (1.200, 6.200) **
CLO	1.048 (0.674, 1.628)	1.629 (0.759, 3.497)	0.502 (0.226, 1.118)
THM	1.056 (0.710, 1.569)	1.048 (0.582, 1.888)	1.070 (0.507, 2.258)
DNT	0.858 (0.623, 1.183)	1.005 (0.604, 1.672)	0.587 (0.296, 1.163)
NIT	1.318 (0.804, 2.161)	2.011 (0.930, 4.347)	0.973 (0.443, 2.134)
SFX	0.881 (0.644, 1.206)	0.631 (0.398, 1.000)	0.587 (0.286, 1.205)
NACE	1.620 (0.969, 2.706)	1.185 (0.524, 2.679)	5.692 (1.910, 16.958) ***
NTHM	1.065 (0.759, 1.493)	1.199 (0.672, 2.139)	0.759 (0.425, 1.357)
ACE	0.946 (0.787, 1.136)	0.828 (0.631, 1.086)	1.184 (0.839, 1.671)
THD	1.211 (0.949, 1.546)	1.252 (0.875, 1.792)	1.231 (0.771, 1.967)

Note. ** *p* < 0.01, *** *p* < 0.001. Bold values indicate statistically significant effects (*p* < 0.05).

**Table 4:** Odds ratios (ORs) and 95% confidence intervals (CIs) of urinary NEO concentrations and hyperactivity dimensions in preschool children.

NEOs	Binary Logistic Regression OR (Hyperactivity, 95% CI)		
	Total	Boys	Girls
IMI	1.692 (1.197, 2.392) ***	1.834 (1.139, 2.954) *	1.961 (1.070, 3.593) *
CLO	0.897 (0.590, 1.362)	0.770 (0.405, 1.467)	1.164 (0.574, 2.360)
THM	1.178 (0.813, 1.707)	1.592 (0.949, 2.671)	0.672 (0.337, 1.344)
DNT	0.837 (0.621, 1.128)	0.858 (0.586, 1.256)	0.746 (0.405, 1.375)
NIT	1.187 (0.761, 1.851)	1.426 (0.766, 2.652)	0.897 (0.470, 1.713)
SFX	1.101 (0.838, 1.445)	1.095 (0.758, 1.583)	0.869 (0.522, 1.447)
NACE	1.193 (0.741, 1.921)	0.854 (0.419, 1.739)	2.567 (1.189, 5.542) *
NTHM	1.467 (1.118, 1.926) ***	1.513 (1.042, 2.196) *	1.474 (0.944, 2.300)
ACE	1.060 (0.889, 1.262)	1.119 (0.881, 1.421)	0.964 (0.700, 1.326)
THD	0.958 (0.768, 1.195)	1.014 (0.755, 1.363)	0.758 (0.503, 1.140)

Note. * *p* < 0.05, *** *p* < 0.001. Bold values indicate statistically significant effects (*p* < 0.05).

**Table 5:** Odds ratios (ORs) and 95% confidence intervals (CIs) of urinary NEO concentrations and total difficulties dimensions in preschool children.

NEOs	Binary Logistic Regression OR (Total Difficulties, 95% CI)		
	Total	Boys	Girls
IMI	1.616 (1.124, 2.323) **	1.482 (0.916, 2.398)	2.223 (1.101, 4.488) *
CLO	0.823 (0.537, 1.261)	0.718 (0.369, 1.398)	0.688 (0.324, 1.460)
THM	0.852 (0.570, 1.273)	1.076 (0.632, 1.834)	0.572 (0.257, 1.275)
DNT	1.010 (0.732, 1.393)	1.172 (0.758, 1.811)	0.730 (0.380, 1.403)
NIT	1.054 (0.663, 1.674)	1.017 (0.533, 1.941)	1.006 (0.485, 2.084)
SFX	1.095 (0.821, 1.460)	1.010 (0.687, 1.486)	0.858 (0.458, 1.604)
NACE	1.783 (1.101, 2.889) *	1.860 (0.929, 3.721)	3.512 (1.402, 8.796) **
NTHM	1.249 (0.928, 1.681)	1.605 (1.078, 2.390) *	0.924 (0.534, 1.602)
ACE	0.988 (0.821, 1.190)	0.919 (0.725, 1.165)	1.111 (0.780, 1.582)
THD	1.119 (0.888, 1.411)	1.205 (0.892, 1.628)	1.195 (0.761, 1.877)

Note. * *p* < 0.05, ** *p* < 0.01. Bold values indicate statistically significant effects (*p* < 0.05).

## Data Availability

The raw data supporting the conclusions of this article are not publicly available due to ethical review restrictions and participant confidentiality requirements but can be provided by the corresponding authors upon request.

## Abbreviations

The following abbreviations are used in this manuscript:

SDQ	The Strengths and Difficulties Questionnaire
NEOs	Neonicotinoid Insecticides
IMI	Imidacloprid
CLO	Clothianidin
THM	Thiamethoxam
DNT	Dinotefuran
NIT	Nitenpyram
SFX	Sulfoxaflor
ACE	Acetamiprid
THD	Thiacloprid
FLO	Flonicamid
6-CINA	6-Chloronicotinic acid
NACE	N-Desmethyl-Acetamiprid
NTHM	N-Desmethyl-Thiamethoxam
WQS	Weighted Quantile Sum
BKMR	Bayesian Kernel Machine Regression
OR	Odds Ratio
CIs	Confidence Intervals
DAG	Directed Acyclic Graph
Cr	Creatinine
RPF	Relative Potency Factor
nAChRs	Nicotinic Acetylcholine Receptors

## Appendix A

**Table A1:** HPLC-MS/MS analytical parameters for NEOs.

Compound	CAS-Number	Elemental Composition	Diagnostic ion	Transitions (*m*/*z*)	Collision Energy (Ev)	Tube Lens Offset Voltage (v)	RT Retention Time (min)
IMI	138261-41-3	C_9_H_10_ClN_5_O_2_	ESI+	256.08/209.12	12	38	3.57
				256.08/174.98	16	38	3.57
CLO	210880-92-5	C_6_H_8_ClN_5_O_2_S	ESI+	250.03/168.87	12	4	3.46
				250.03/131.87	14	4	3.46
THM	153719-23-4	C_8_H_10_ClN_5_O_3_S	ESI+	292.05/210.94	14	24	3.28
				292.05/131.92	18	24	3.28
ACE	135410-20-7	C_10_H_11_ClN_4_	ESI+	223.03/125.88	24	40	3.66
				223.03/55.92	14	42	3.66
THD	111988-49-9	C_10_H_9_ClN_4_S	ESI+	252.99/125.9	18	52	3.84
				252.99/90.07	40	52	3.84
DNT	165252–70–0	C_7_H_14_N_4_O_3_	ESI+	203.07/128.93	12	4	2.76
				203.07/113.26	10	4	2.76
NIT	150824-47-8	C_11_H_15_ClN_4_O_2_	ESI+	271.12/125.97	28	2	3.44
				271.12/129.71	12	2	3.44
SFX	946578-00-3	C_10_H_11_F_3_N_3_OS	ESI+	278.01/173.97	8	28	3.85
				278.01/153.94	28	28	3.85
FLO	158062-67-0	C_9_H_6_F_3_N_3_O	ESI+	230.07/203.00	16	22	3.32
				230.07/173.98	18	22	3.32
6-ClNA	5326-23-8	C_6_H_4_ClNO_2_	ESI+	158.02/121.92	16	22	3.48
				158.02/77.96	22	22	3.48
N-ACE	190604-92-3	C_9_H_9_ClN_4_	ESI+	209.02/125.89	18	16	3.49
				209.02/90.05	32	26	3.49
N-THM	171103-04-1	C_7_H_8_ClN_5_O_3_S	ESI+	278.03/173.99	10	30	3.9
				278.03/104.87	10	30	3.9

**Table A2:** The chronic reference dose and relative toxic effect factors of neonicotinoids.

NNIs	cRFD (mg/kg/Day)	RPF
IMI	0.080	1.000
CLO	0.098	0.820
THM	0.012	6.670
DNT	1.000	0.080
NIT	-	0.110
SFX	0.050	1.600
NACE	-	1.130
NTHM	-	6.670
ACE	0.071	1.130
THD	0.004	20.000

**Table A3:** WQS regression index between urinary neonicotinoid concentrations and various dimensions of neurobehavioral development in preschool children (All people, *n* = 506).

Outcomes	*β*	*p*
Emotional problem	0.970	*p* < 0.001
Hyperactivity	0.961	*p* < 0.001
Total difficulties	2.138	*p* < 0.001

**Table A4:** WQS regression weights between urinary neonicotinoid concentrations and emotional symptoms in preschool children.

Mix Name	Mean Weight (Emotional Symptoms)		
	Total	Boys	Girls
IMI	46.44%	47.67%	42.42%
CLO	0.55%	2.14%	3.34%
THM	7.75%	2.30%	2.51%
DNT	4.43%	0.14%	1.97%
NIT	1.57%	0.50%	5.98%
SFX	0.14%	2.93%	0.26%
NACE	12.27%	8.70%	20.50%
NTHM	16.02%	29.84%	7.81%
ACE	6.19%	2.01%	2.53%
THD	4.63%	3.77%	12.67%

**Table A5:** WQS regression weights between urinary neonicotinoid concentrations and hyperactivity in preschool children.

Mix Name	Mean Weight (Hyperactivity)		
	Total	Boys	Girls
IMI	34.88%	37.43%	31.64%
CLO	0.71%	0.14%	3.00%
THM	1.87%	1.19%	2.91%
DNT	0.83%	0.11%	1.47%
NIT	4.34%	0.16%	10.61%
SFX	9.97%	8.81%	1.31%
NACE	21.98%	18.27%	12.97%
NTHM	13.64%	21.00%	30.29%
ACE	9.50%	11.42%	1.16%
THD	2.28%	1.47%	4.64%

**Table A6:** WQS regression weights between urinary neonicotinoid concentrations and total difficulties in preschool children.

Mix Name	Mean Weight (Total Difficulties)		
	Total	Boys	Girls
IMI	41.57%	38.17%	31.77%
CLO	1.36%	0.91%	4.21%
THM	7.46%	4.46%	2.60%
DNT	5.10%	0.06%	4.40%
NIT	1.18%	0.32%	5.31%
SFX	1.97%	12.66%	1.13%
NACE	11.79%	9.11%	17.03%
NTHM	12.35%	23.16%	25.37%
ACE	13.61%	10.15%	2.11%
THD	3.60%	1.00%	6.07%
